# Sintilimab-Induced Diabetic Ketoacidosis in a Patient with Radiation and Multichemorefractory Penile Cancer: A Case Report and Literature Review

**DOI:** 10.3390/curroncol29110632

**Published:** 2022-10-25

**Authors:** Chuan Lv, Can Wu, Yan Zhang, Wendong Li, Xuesong Wang, Li Liang

**Affiliations:** 1 Department of Endocrinology, People’s Hospital of China Medical University, The People’s Hospital of Liaoning Province, Shenyang 110016, China; 2 Endoscopy Department, The First Affiliated Hospital of China Medical University, Shenyang 110001, China; 3 Department of Ophthalmology, Shenyang Tenth People’s Hospital, Shenyang 110044, China; 4 Radiology Department, People’s Hospital of China Medical University, Shenyang 110016, China; 5 Department of Urology, People’s Hospital of China Medical University, Shenyang 110016, China

**Keywords:** autoimmune diabetes, PD-1 inhibitor, sintilimab, penile carcinoma

## Abstract

Penile squamous cell carcinoma (PSCC) is a rare disease. The treatment options for advanced penile cancer are often limited, and the prognosis remains poor. We reported a 52-year-old male recurrent and metastatic PSCC patient with high PD-L1 expression (90%) and TMB (14.4 muts/Mb). He had undergone penectomy, bilateral inguinal lymph node dissection, and excision of the abdominal wall mass. Despite cisplatin-based concurrent chemoradiotherapy and sequential chemotherapy with docetaxel plus cisplatin then being carried out, the carcinoma still progressed. The patient then obtained progression-free survival with continuous sintilimab, although he experienced the new onset of ICI-induced diabetes after 24 cycles of sintilimab and required sustained insulin treatment. He had negative type 1 diabetes-associated autoantibodies and the susceptible HLA genotype DR3-DQ2 haplotype. This is the first patient with radiation and multichemorefractory PSCC who has obtained the remarkable anti-tumor effect of partial regression exceeding 32 months during continuous sintilimab and anlotinib treatment.

## 1. Background

In recent years, immune checkpoint inhibitors (ICIs), a novel class of drugs for tumor immunotherapy, have emerged as a therapeutic approach against various malignancies, although some patients do not respond to ICIs [1,2]. ICIs can activate the autoimmune response to tumor cells and block the immune escape of tumors by inhibiting negative immune regulation proteins, including cytotoxic T cell-associated antigen 4 (CTLA4), programmed cell death protein 1 (PD-1), and programmed cell death protein ligand 1 (PD-L1) [3]. However, the inhibition of immune checkpoints sometimes also damages self-tissues, causing immune-related adverse events, including dermatological, pulmonary, gastrointestinal, cardiovascular, and endocrine system-related side effects [4]. Type 1 diabetes mellitus (T1DM) caused by PD-1/programmed cell death ligand-1 inhibitors is rare and seldom reported, especially in patients with penile cancer [5]. Sintilimab (Innovent Biologics, Suzhou, China), a type of PD-1 antibody, was included in the 2019 edition of The Lymphoma Diagnosis and Treatment Guide of The Chinese Society of Clinical Oncology [6]. It has been approved to treat various advanced tumors, such as non-small cell lung cancer, esophageal cancer, gastric cancer, hepatocellular carcinoma, and Hodgkin’s lymphoma. Here, we report the first case of new-onset autoimmune diabetes in a patient who received sintilimab plus anlotinib to treat radiation and multichemorefractory penile squamous cell carcinoma (PSCC).

## 2. Case Report

**Figure 1:**
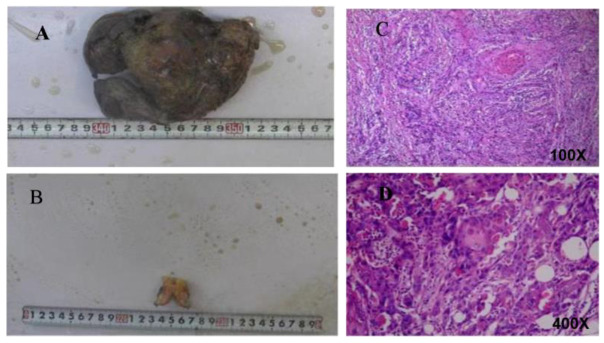
Pathology images of postoperative tissues after recurrence. Picture of penis tumor tissue (**A**) and abdominal wall tumor tissue (**B**); H and E staining of penis tumor tissue sample (**C**) and abdominal wall tumor tissue sample (**D**).

A 52-year-old man was pathologically diagnosed with squamous cell carcinoma of the penis and inguinal lymph node metastases by biopsy of a glans penis mass and the right inguinal lymph node in December 2017. Fluorodeoxyglucose-positron emission tomography/computed tomography scanning showed a hypermetabolic mass at the penis root below the bulb of the urethra and the adjacent scrotum. Then, he underwent partial amputation of the penis and bilateral inguinal lymph node dissection. In April 2018, he underwent concurrent chemotherapy (cisplatin (40 mg/m^2^) for 5 weeks) and radiotherapy (2.0 Gy/fraction, total 60 Gy). In May 2018, a sequential chemotherapy regimen (docetaxel (120 mg/m^2^ day 1) plus cisplatin (40 mg/m^2^ day 1–3), 4 cycles) was prescribed for him. In November 2018, percutaneous cystostomy was performed because of dysuria. Nearly two months later, the PSCC relapsed, with metastases in the left inguinal and iliac lymph nodes, right pelvic wall lymph nodes, and the abdominal skin on the right side of the external orifice of cystostomy tube. Due to severe pain, he underwent total penectomy and excision of the abdominal wall mass, which were pathologically proven to be squamous cell carcinoma (Figure 1). Unfortunately, less than five months after the second surgery, he became physically weak, with an Eastern Cooperative Oncology Group (ECOG) score of 4. Abdominal computed tomography (CT) showed that the carcinoma had spread to the perineum area with infection, with metastases in bilateral inguinal and pelvic wall lymph nodes and the left external iliac vein being invaded.

**Figure 2:**
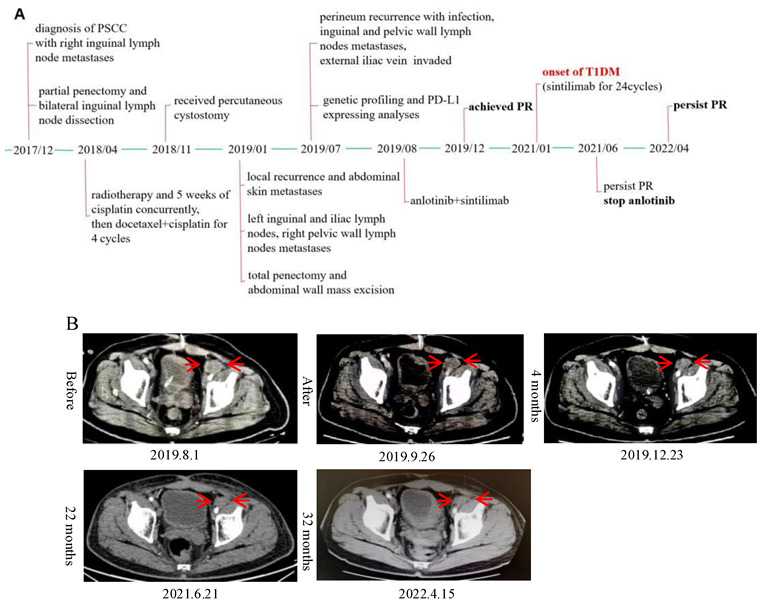
(**A**) Timeline of the patient’s therapy and the effects of therapy. Imaging showing metastasis of pelvic lymph nodes. (**B**) Contrast-enhanced abdominal computed tomography assessments of pelvic wall tumor nodule during each period. Red arrows indicate the tumors.

Subsequently, next-generation sequencing and PD-L1 immunohistochemistry were performed to seek potential therapeutic options. The results indicated positive PD-L1 expression (90%), with a combined positive score (CPS) of 110 that was microsatellite stable and a tumor mutational burden (TMB) of 14.4 (muts/Mb). Genomic analysis of the tissue sample taken from the tumor showed a 2.3 times amplification of the epidermal growth factor receptor. The patient was, then, started on anlotinib treatment (12 mg/day for two weeks and then stopped for one week) in August 2019. Fourteen days later, he voluntarily started to receive 200 mg of sintilimab every 3 weeks. He signed informed consent for the off-label use of sintilimab. Several days later, his physical strength and mental status improved significantly. Four months after receiving sintilimab, the evaluation showed sustained response (Figure 2). During the following 32 months, the patient’s ECOG score was 1.

Nevertheless, in January 2021, after 24 cycles of sintilimab, he complained of polyuria, thirst, and fatigue. Two days later, he was admitted to the emergency department for nausea and vomiting. No abnormalities were found on physical examination. Blood tests showed that his instant plasma glucose was as high as 24.27 mmol/L, with an HbA1c value of 7.3%. Arterial blood gas analysis revealed that he had metabolic acidosis, with an arterial pH of 7.329, serum bicarbonate of 20.2 mmol/L, and lactate of 1.3 mmol/L. The urine ketone body determination was positive. Thus, diabetic ketoacidosis was diagnosed. He initially received maintenance water, insulin therapy delivered by micropump, acid–base and electrolyte balance, and supportive treatment to correct the acidosis. Then, further blood assessment showed the exhaustion of his islet beta cell function, with a fasting C-peptide value of 0.13 ng/mL, which further decreased to 0.02 ng/mL within one month. There was no medical history or family history of hyperglycemia, infection, autoimmune diseases, endocrine diseases, other systemic and hereditary diseases that could cause hyperglycemia. Consequently, he was diagnosed with sintilimab-induced autoimmune diabetes. However, the anti-glutamic acid decarboxylase antibody (GADA), anti-islet cell antibody (ICA), protein tyrosinephosphatase antibody (IA-2A), and anti-insulin antibody (IAA) were all negative. In addition, human leucocyte antigen (HLA) class I and II type analysis revealed that he had the T1DM-sensitive genotype DRB1*0301-DQB1*0201. He was also identified as having five T1DM risk loci, including rs689 in INS, rs2476601 in PTPN22, rs1990760 in IFIH1, rs3757247 in BACH2, and rs11202303 in UBASH3A. For other endocrine function assessments, thyroid hormones showed that the patient’s serum free triiodothyronine (FT3) and free thyroxine were normal. Moreover, the levels of anterior pituitary hormones and their regulated hormones were all normal.

Thereafter, he received continuous subcutaneous insulin therapy via an insulin pump during his hospital stay. Before discharge, insulin treatment was adjusted daily to the dose of once-daily insulin glargine (long-acting insulin, 10 units) plus thrice-daily prandial insulin aspart (fast-acting insulin, 5 units). He presented with progression-free carcinoma as well as moderate glycemic control and experienced no other side effects during follow-up visits. Thus, anlotinib treatment was stopped in June 2021, but sintilimab administration was continued.

## 3. Discussion

Penile cancer is a rare condition that mostly affects men in their sixth decade of life. The most common histology is squamous cell carcinoma, which is primarily treated by surgical resection. A timely multidisciplinary treatment approach at an experienced center is critical for improving outcomes. Unfortunately, advanced penile cancer represents a significant challenge in clinical practice, as treatment options are often limited, and prognosis remains poor [7,8]. Thus, novel molecular and immunotherapeutic targets are actively being sought [7,9]. Targeted therapies and ICIs are expected to play a role in advanced penile carcinoma [8,10]. However, there are no published randomized clinical trials on patients with distant metastatic disease who have already received standard chemotherapy, due to the rarity of PSCC [8].

Several ICIs, such as nivolumab [11,12,13], atezolizumab [13], pembrolizmab [14,15], cemiplimab [16], and toripalimab [17], have been experimentally utilized as single second-line agents to treat metastatic penile cancer. Comfortingly, most of the patients in these case reports presented with a partial response or a near-complete response. Hu et al. [18] reported a recurrent PSCC patient with medium PD-L1 expression and low TMB who obtained complete response after multimodal therapy that included surgical resection, adjuvant chemotherapy, and continuous sintilimab. It was also recently reported that immunotherapy combined with chemotherapy could have good therapeutic effects in advanced PSCC patients [19,20]. Our patient was first reported as obtaining a partial response to combined treatment with sintilimab plus the target drug, with progression-free survival exceeding 32 months. Thus, immunotherapy may play a pivotal role in the above therapies. Furthermore, we conducted a comprehensive literature analysis to evaluate whether immunotherapy can benefit patients with advanced penile SCC (Table 1). Thirteen patients have been reported as obtaining a partial or complete response to immunotherapy. The positive ratio of PD-L1 expression was 90.9%, with 66.7% being microsatellite stable and 66.7% with high TMB. Thus, positive PD-L1 and high TMB could be potential biomarkers for ICI treatment in PSCC. However, evidence from several case reports is limited.

ICIs create the hope for longer survival and improved quality of life in patients with advanced malignancies. However, the excessive activation of immune cells may also cause immune-related adverse events (irAEs), resulting in disorders of multiple endocrine and non-endocrine organs [21]. T1DM is a rare irAE of PD-1 inhibitors and is expected to increase with the use of ICIs in clinical practice. Here, we report the first case of diabetic ketoacidosis caused by new-onset auto-immune diabetes during sintilimab plus anlotinib therapy for recurrent metastatic PSCC after resistance to concurrent chemoradiation. Our present case was consistent with previous reports in which the majority of the patients with auto-immune diabetes presented with diabetic ketoacidosis (50.2%), with the onset of diabetes ranging from 5 to 880 days after the first dose of ICIs [22,23]. Our patient’s HbA1c level was high, 7.3%, supporting the view that some degree of hyperglycemia, or significant hyperglycemia during a shorter period, had been present prior to its acute presentation [24]. ICI-induced diabetes sometimes resembles fulminant diabetes, and routine blood glucose monitorization may not detect or predict its occurrence. Thus, diabetes education, routine self-monitoring of blood glucose levels, and determination of serum glycated albumin and C-peptide during each treatment cycle may be beneficial for the early identification of new-onset T1DM.

Although the physiopathology of auto-immune diabetes associated with ICIs is unknown, the activation of autoreactive T cells caused by a reduction of PD-1 might result in an autoimmune response against islet cells. Previous reviews have shown that the most commonly positive islet autoantibody is GAD65, which is only detectable in about 51% of patients [22,24]. Our patient presented with negative diabetes-related antibodies, supporting the view that the presence of islet autoantibodies is not an absolute requirement for the diagnosis of ICIs-associated diabetes. In addition to islet autoantibodies, certain HLA genotypes are known to predispose an individual to type 1 diabetes. DQA1*0301-DQB1*0302/DQA1*0501-DQB1*0201 has been identified as a high-risk genotype in Caucasians. In Asians, the haplotypes DRB1*0405-DQB1*0401, DRB1*0802-DQB1*0302 and DRB1*0901-DQB1*0303, and the DRB1*0802-DQB1*0302 genotype, have been classified as resulting in susceptibility to acute-onset and slowly progressive T1DM. In contrast, only DRB1*0405-DQB1*0401 is associated with fulminant type 1 diabetes. In addition, an association of B*4002 with fulminant type 1 diabetes has also been identified [25]. Pociot et al. [26] proposed that the primary risk factor for β-cell autoimmunity is genetic, mainly occurring in individuals with either the HLA-DR3-DQ2 and/or HLA-DR4-DQ8 haplotypes. The frequency of HLA-DR4 was reported to be more than 60% in ICI-induced T1DM and was much higher than that in conventional T1DM, where the frequency of HLA-DR3 was about 30% [21,27]. HLA typing of our patient revealed the DR3-DQ2 haplotype, which is a susceptible genotype, supporting the finding that there appears to be a cohort of people who are at risk of HLA that develops into ICI-associated diabetes [28].

## 4. Conclusions

To our knowledge, this is the first case describing a patient who had radiation and multichemorefractory PSCC who was treated with sintilimab plus anlotinib and achieved progression-free survival exceeding 32 months. Although ICI-induced diabetes persisted, and required sustained treatment with insulin, this may provide support for single immunotherapy as a new option to maximize the benefits for advanced penile cancer patients.

## Figures and Tables

**Table 1:** Clinical features of reported cases.

Author	Age	PD-L1 Status	MSI Status	TMB	Outcome
Baweja et al. [11] (2021)	47	TPS: 90%	MSI-high	High (24 Muts/Mb)	12-month follow up, PR
Trafalis et al. [12] (2018)	47	TPS: ≈10%	Stable	High	6-month follow up, PR
Hui et al. [13] (2019)	64	UK	UK	UK	6-month follow up, Stable
Hui et al. [13] (2019)	79	UK	UK	UK	24-month follow up, CR
Chahoud et al. [14] (2020)	64	UK	UK	High (14 Muts/Mb)	38-month follow up, CR
Chahoud et al. [14] (2020)	85	CPS: 130	Stable	Low (3 Muts/Mb)	18-month follow up, PR
Hahn et al. [15] (2021)	76	TPS: 10%	MSI-high	UK	38.7-month follow up, PR
Hahn et al. [15] (2021)	72	TPS: 80%	Stable	UK	8.3-month follow up, PD
Hahn et al. [15] (2021)	66	TPS: 1%	Stable	UK	3.8-month follow up, PD,
Denis et al. [16] (2021)	75	TPS: >95%	UK	UK	15.1-month follow up, CR
Su et al. [17] (2020)	46	TPS: ≥10%	Stable	High (8.87 Muts/Mb)	10.5-month follow up, CR
Hu et al. [18] (2021)	49	TPS: 20~30%	Stable	Low (2.25 Muts/Mb)	19-month follow up, CR
Li et al. [19] (2022)	76	TPS: ≈10%	UK	UK	5-month follow up, CR
Mei et al. [20] (2022)	63	TPS: 50~60%	Stable	High (17.95 Muts/Mb)	28-month follow up, PR
Mei et al. [20] (2022)	39	TPS: <1%	Stable	Low (0 Muts/Mb)	24-month follow up, CR
Present case (2022)	52	TPS: 90%	Stable	High (14.40 Muts/Mb)	32-month follow up, PR

MSI, microsatellite instability; TMB, tumor mutation burden; PR, partial response; CR, complete response; PD, progression of disease; UK, unknown; TPS, tumor proportion score; CPS, combined positive score.

## Data Availability

The raw data presented in the study is included in the article. Further inquiries can be directed to the corresponding author.
